# Assessing the perioperative gain of weight (Δweight) as a determinant of morbidity after kidney transplantation: a retrospective exploratory study

**DOI:** 10.1038/s41598-024-63950-8

**Published:** 2024-06-11

**Authors:** Beatriz Barberá Carbonell, Tobias Zingg, Maurice Matter, Gaëtan-Romain Joliat, David Martin, Manuel Pascual, Nicolas Demartines, Dela Golshayan, Luis Cano, Ismail Labgaa

**Affiliations:** 1 Department of Visceral Surgery, Lausanne University Hospital (CHUV), Rue du Bugnon 46, CH-1011 Lausanne, Switzerland; 2 Faculty of Biology and Medicine (FBM), University of Lausanne (UNIL), Lausanne, Switzerland; 3 Graduate School of Health Sciences, University of Bern, Bern, Switzerland; 4 Transplantation Center, Lausanne University Hospital (CHUV), Lausanne, Switzerland; 5 Nutrition Metabolism and Cancer, INSERM, University of Rennes, INRAE, CHU Pontchaillou, UMR 1241 NUMECAN, Rennes, France

**Keywords:** Complications, Prediction, Biomarkers, Transplant, Outcomes, Biomarkers, Medical research, Nephrology

## Abstract

Kidney transplantation (KT) is associated with a substantial risk of postoperative complications (POC) for which performant predictors are lacking. Data showed that a perioperative gain of weight (ΔWeight) was associated with higher risk of POC, but it remains unexplored in KT. This retrospective study aimed to investigate the association between ΔWeight and POC after KT. ΔWeight was calculated on postoperative day (POD) 2. POC were graded according to the Dindo-Clavien classification. Primary endpoint was overall POC. A total of 242 patients were included and 174 (71.9%) complications were reported. Patients showed a rapid gain of weight after KT. Mean ΔWeight was 7.83 kg (± 3.20) compared to 5.3 kg (± 3.56) in patients with and without complication, respectively (*p* = 0.0005). ΔWeight showed an accuracy of 0.74 for overall POC. A cut-off of 8.5 kg was determined. ΔWeight ≥ 8.5 kg was identified as an independent predictor of overall POC on multivariable analysis (OR 2.04; 95% CI 1.08–3.84; *p* = 0.025). ΔWeight ≥ 8.5 kg appeared as an independent predictor of POC after KT. These results stress the need to monitor weight in KT and to further investigate this surrogate with future studies assessing its clinical relevance.

## Introduction

Kidney transplantation (KT) is acknowledged as the treatment of choice for end-stage renal disease (ESRD), but its access is dramatically limited by organs shortage and costs^1^.

Important efforts have been pursued both on medical and surgical perspectives, resulting in substantial improvements in patient and graft survival. Nonetheless, KT remains associated with a non-negligeable risk of morbidity^2,3^. Few studies determined overall postoperative complications (POC) as their primary endpoint. Most studies targeted specific adverse outcomes after KT, such as mortality, graft survival, delayed graft function (DGF) or infectious complications^4–7^. Likewise, studies investigating the value of markers like lab tests to predict adverse outcomes are scant. Conversely, novel predictors such as dynamic surrogate markers of the stress response have been identified in other types of surgery^8,9^. Among them, the perioperative fluctuation of weight (ΔWeight) appeared as a valuable marker to anticipate POC after digestive surgery^9–11^. Considering the intensity of the perioperative physiological stress response and fluid management, ΔWeight appears as an interesting candidate to predict POC in KT. In addition, it displays other important and valuable characteristics: early indicative, inexpensive, readily available and reproducible^12,13^.

This study aimed to explore the potential contribution of ΔWeight to predict adverse events after KT.

## Results

A total of 327 patients underwent KT during the period of inclusion. After exclusion of patients without signed consent (n = 30), pediatric patients (n = 8), and patients with missing data (n = 47), a total of 242 patients met eligible criteria and were included for final analyses. Characteristics of this cohort are detailed in Table 1.

**Table 1:** characteristics of the cohort.

	Value Mean (± SD) or n (%)		*p*-value
	ΔWeight < 8.5 kg (n = 161)	ΔWeight ≥ 8.5 kg (n = 81)	
Age (years)	52.6 (15.2)	55.5 (13.6	0.22
Gender (Female)	45 (28)	33 (40.7)	0.06
ASA score (≥ 3)	143 (88.8)	73 (90.1)	0.929
Smoking	43 (26.7)	29 (35.8)	0.18
Diabetes	29 (18)	15 (18.5)	0.99
Hypertension	140 (87)	72 (88.9)	0.82
Cirrhosis	2 (1.2)	0 (0)	0.79
BMI (≥ 25 kg/m^2^)	25.6 (4.85)	25.8 (4.43)	0.804
Type of graft (deceased-donor)	87 (54)	61 (75.3)	< 0.01
Immunosuppression	27 (16.8)	15 (18.5)	0.87
Surgery duration (min)	167 (59.9)	171 (59.2)	0.506
Preoperative albuminemia (g/L)	41.68 (5.04)	40.36 (6.69)	0.21

SD: Standard deviation; ASA: American society of anesthesiologists; BMI: Body mass index.

### Perioperative change of weight (Δweight)

**Figure 1:**
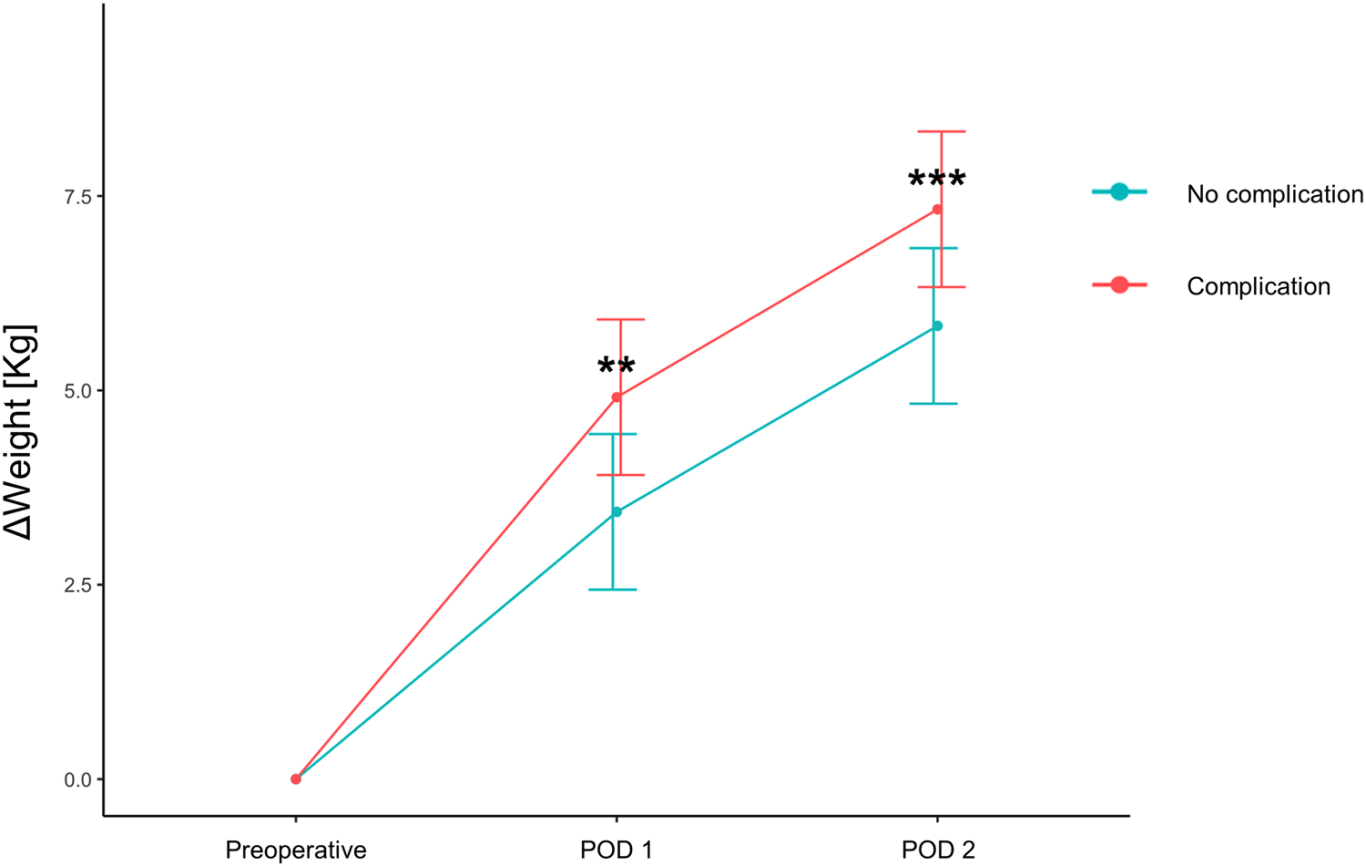
Perioperative profile of weight change. Curves show mean values of weight change. Patients without and with postoperative complications are represented by green and red curves, respectively. Bars show standard error of the mean. ** *p* = 0.002. *** *p* = 0.0005. POD: Postoperative day.

The perioperative course of patients was typically characterized by a rapid gain of weight on POD 1 and 2 (Fig. 1). This gain of weight was more pronounced in patients developing complications. ΔWeight calculated on POD 2 showed a mean value of 7.83 (± 3.20) Kg among patients with complications, compared to 5.3 (± 3.56) Kg in patients without complications (*p* = 0.0005).

### Accuracy, threshold, predictive values and application of Δweight

**Figure 2:**
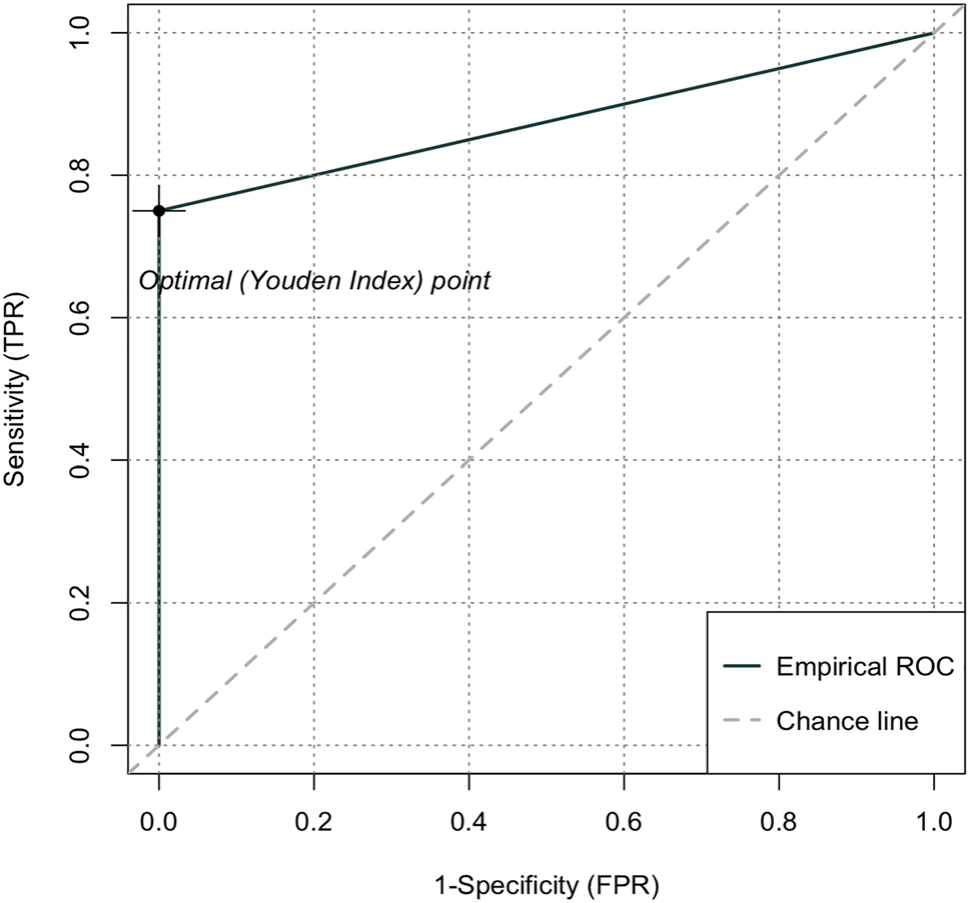
ROC curve of ΔWeight for overall complications. Receiver operating characteristic (ROC) curve of ΔWeight for overall complications. Area under the curve (AUC) yielded an accuracy of 0.74 (*p* = 0.0002).

ROC curve of ΔWeight for the prediction of overall complications showed an area under the curve (AUC) of 0.74 (*p* = 0.0002) (Fig. 2). The use of the Youden index established a cut-off of 8.5 kg, yielding positive and negative predictive values of 100% and 75%, respectively.

Applying this cut-of allowed distinguishing patients at higher risk of adverse events. Patients with ΔWeight ≥ 8.5 kg showed higher rates of minor complications (80.2% vs. 61.5%, *p* = 0.003), overall complications (85.2% vs. 65.2%, *p* = 0.001), higher CCI (29.6 vs. 20.9, *p* = 0.01) and longer LoS (29.9 vs. 22.7 days, *p* = 0.004), in comparison to patients with ΔWeight < 8.5 kg (Table 2).

**Table 2:** Outcomes.

	Whole cohort (n = 242)	< 8.5 kg (n = 161)	≥ 8.5 kg (n = 81)	*p*-value
Minor complications (< grade III)	164 (67.8)	99 (61.5)	65 (80.2)	0.003
Major complications (≥ grade III)	83 (34.3)	51 (31.7)	32 (39.5)	0.28
Overall complications	174 (71.9)	105 (65.2)	69 (85.2)	0.001
CCI	26.2 (38.92)	20.9 (36.2)	29.6 (18.8)	0.01
LoS (days)	25.11 (9)	22.7 (9)	29.9 (8)	0.004
Delayed graft function (DGF)	71 (29.33)	42 (26.08)	29 (35.80)	0.156
Rejection	29 (11.98)	19 (11.80)	10 (12.34)	1
Post-transplant hemodialysis	33 (13.63)	19 (11.80)	14 (17.28)	0.32
Infectious complications	53 (21.90)	30 (18.63)	23 (28.39)	0.11
Cardiovascular complications	12 (4.95)	8 (4.96)	4 (4.93)	1
Glucocorticoid-induced diabetes	27 (11.15)	18 (11.18)	9 (11.11)	1
Thromboembolic complications	13 (5.37)	12 (7.45)	1 (1.23)	0.08
Lymphocele	10 (4.13)	5 (3.10)	5 (6.17)	0.43
Surgical site infection (SSI)	4 (1.65)	1 (0.62)	3 (3.70)	0.21
Evisceration/incisional hernia	5 (2.06)	4 (2.48)	1 (1.23)	0.86
Drug-induced complications	8 (3.30)	5 (3.10)	3 (3.70)	1
Re-operation	29 (11.98)	15 (9.31)	14 (17.28)	0.11
ΔCreat (µmol/L)	232 (218)	267 (211)	163 (218)	< 0.001

Data provide the number of events with percentages (%), or mean values with standard deviation (SD). CCI: Comprehensive complication index; LoS: Length of stay.

Uni- and multi-variable analysis integrating multiple potential confounding factors identified 4 independent predictors of complications: hypertension (OR 7.92; 95% CI 1.77–35.38; *p* < 0.001), deceased donor (OR 3.05; 95% CI 1.65–5.64; *p* = 0.008), ΔWeight ≥ 8.5 kg (OR 2.04; 95% CI 1.08–3.84; *p* = 0.025) and ΔCreat (OR 0.99; 95% CI 0.996–0.999; *p* = 0.046) (Table 3). For severe complications, multivariable analysis identified duration of surgery (OR 1.01; 95% CI 1.00–1.01; *p* = 0.042) and ΔCreat (OR 1.00; 95% CI 1.00–1.00; *p* = 0.03) as independent predictive factors (Supplementary Table 1).

**Table 3:** Uni- and multi-variable analysis for overall complications.

	Univariable			Multivariable		
	OR	95% CI	*p*-value	OR	95% CI	*p*-value
Age (years)	1.02	1.00–1.04	0.109			
Gender (female)	1.80	0.95–3.41	0.065			
ASA score (< 2)	0.34	0.15–0.78	0.012	0.46	0.14–1.14	0.055
Smoking	1.93	0.99–3.76	0.046	0.54	0.96–1.12	0.134
Diabetes (no diabetes)	0.71	0.33–1.53	0.373			
Hypertension	6.33	1.46–27.35	0.002	7.92	1.77–35.38	< 0.001
BMI (kg/m^2^)	1.01	0.96–1.08	0.627			
Type of graft (deceased-donor)	3.17	1.78–5.67	< 0.001	3.05	1.65–5.64	0.008
Duration of surgery (min)	1.00	1.00–1.01	0.504			
ΔWeight (≥ 8.5 kg)	3.07	1.53–3.14	< 0.001	2.04	1.08–3.84	0.025
ΔCreat (µmol/L)	0.99	0.996–0.998	< 0.001	0.99	0.996–0.999	0.046

OR: Odd ratio; CI: Confidence interval; ASA: American society of anesthesiologists; BMI: Body mass index.

## Discussion

Findings of the present study suggested that a perioperative gain of weight ≥ 8.5 kg on POD 2 (ΔWeight) was associated with an increased risk of adverse events and identified ΔWeight as an independent predictor of overall complications after KT.

A first striking observation was the perioperative weight profile (Fig. 1). Regardless of the outcome (i.e. complication or no complication), patients showed a rapid and substantial weight gain after KT. Weight gain was substantially higher than previously reported for other types of major surgery^9,10^. The underlying causes/mechanisms of such response are likely multifactorial: capillary leak, inflammatory response, metabolites releases, hematocrit and/or albuminemia decreases, intra- and postoperative fluid management. Regarding the latter, our group already investigated the correlation between the volume of intraoperative fluid administration and ΔWeight in liver surgery and a poor correlation was observed, suggesting that intraoperative fluid was not the main determinant of Δweight^9^. However, the targeted central venous pressure is higher in KT than in other types of surgery; this could be an important difference. Although fascinating, this question was not the target of the present study and requires more comprehensive prospective data to be answered. With this perspective, it will be pivotal to investigate factors involved in surgical stress response^14,15^. ΔWeight is unlikely only attributable to suboptimal graft function and/or fluid overload.

Another obvious question is the potential impact of ΔWeight in clinical practice. First, the simple fact of identifying patients at higher risk has a valuable impact for clinicians managing patients after KT. It is however unknown whether specific measures (more restrictive fluid management during the first days after KT or pharmacological interventions) can mitigate ΔWeight with no other side effects. This is a critical point deserving to be investigated by future research.

We thought that graft function may be a confounding factor. To exclude this hypothesis, we integrated ΔCreat -which was also calculated on POD2- in the multivariable model. Interestingly, both ΔWeight and ΔCreat were identified as predictors but they are independent from each other’s.

ΔWeight is a recent surrogate marker for outcome after surgery for which data are scarce. Few available studies were conducted in digestive surgery but none in KT. Likewise, studies in KT exploring other markers are rare. Classical biomarkers like C-Reactive Protein (CRP), procalcitonin (PCT), interleukine-6 (IL-6) or ΔAlbumin showed interesting results in abdominal surgery but their contribution remains unclear in KT. In fact, the postoperative immunosuppression required after KT may have an effect on the kinetics of these markers and thereby significantly challenges their value in this setting. This is an important limitation of these surrogate markers, from which ΔWeight does not suffer. Furthermore, ΔWeight displays many precious specificities: early indicative (i.e. already on POD 2), reproducible and inexpensive. One may wonder whether ΔWeight is a predictor of POC or if it simply reflects the consequence of POC. The very early nature of ΔWeight -calculated ≤ 48 h after KT- precludes considering it as a consequence of POC and rather emphasizes its predictive nature.

Limitations of this study include its retrospective, single-center design as well as a modest sample size. As detailed, 47 patients showed missing data; hence, selection bias cannot categorically be excluded. An external validation set was also lacking. On the other hand, stringent criteria were applied for patient selection, to minimize bias.

In summary, the results showed an association between ΔWeight and adverse events after KT, identifying ΔWeight as an independent predictor of overall complications. These findings stress the need to monitor and limit the perioperative weight gain of patients undergoing KT and to intensify research on the topic, particularly to clarify how targeting ΔWeight may improve patient outcomes in KT.

## Methods

### Design of the study

Retrospective study including all consecutive patients undergoing KT at Lausanne University Hospital (CHUV), Switzerland, between January 2015 and March 2021. Informed consent was obtained from all included patients. The study protocol followed TRIPOD guidelines and was approved by the Institutional Review Board of Lausanne University Hospital (CER-VD # 2018–01,987).

### Selection of patients and data collection

Patients > 18 years undergoing KT during the period of inclusion were eligible. To minimize the risk of bias, patients with any missing variables were stringently excluded. Collected variables included demographics, surgical details, perioperative clinical and laboratory parameters, and outcomes. Following KT, all patients were managed according to standard protocols regarding fluid management and immunosuppressive therapy. Briefly, standard immunosuppressive therapy consisted of basiliximab induction followed by tacrolimus, mycophenolate mofetil, and corticosteroids maintenance therapy. Thymoglobulin was administered according to the recipient's immunologic risk.

### ΔWeight

Perioperative weight was measured and recorded by nurses on a daily basis. As previously described, ΔWeight was calculated in kilograms (Kg) according to the following formula: [weight on POD 2 – preoperative weight]^9^. POD 2 was specifically selected as it displays several precious features: (I) lower rates of missing data compared to POD 1, for reasons of hemodynamic monitoring, making weighing challenging, (II) likely late enough to reflect perioperative fluid shifts after KT, and (III) early enough to act as a predictor and not just as a marker of POC. The perioperative variation of serum creatinine (SC) was also obtained on POD 2, defined as ΔCreat and calculated in [µmol/L] according to this formula: [preoperative SC – SC on POD 2].

### Endpoints

Morbidity was the outcome of interest. Postoperative complications were graded according to Dindo-Clavien classification and divided in minor (grade I-II) and major complications (grade ≥ III).

The primary endpoint was overall complications whereas secondary endpoints were major complications (grade III-IV according to Dindo-Clavien), comprehensive complication index (CCI)^16^ and length of stay (LoS). LoS was calculated from day of surgery until discharge, as previously described^17,18^.

Delayed graft function (DGF) was defined as the need for dialysis in the first postoperative week (7 days) and/or the absence of significant decrease in serum creatinine (at least 10% daily) during 3 successive days, in the first week after KT, irrespective of dialysis requirement^19–21^.

### Statistical analysis

All statistical analyses were performed by a biostatistician.

A process of data cleaning was performed to remove non-available data (NAD), using the dplyr package from R version 4. This step ensured the integrity of the data and prevented possible deviations from the results. The optimal cut-off point for ΔWeight was determined by plotting the receiver operating characteristic (ROC) curve. Different sensitivity and specificity values were evaluated, and the point displaying the highest performance was selected. The ROC curve was built using the proc package in R, establishing a threshold that divided patients into two distinct groups based on ΔWeight.

Subsequently, an analysis was performed to assess significant differences between the groups using the cut-off of ΔWeight. Appropriate statistical tests, such as the Student`s *t*-test or chi-square test, were utilized to compare demographic and clinical characteristics between the two groups. All χ2 tests were supervised and corrected, following recommendations by Yates^22^. Univariable logistic regression model was developed by considering all variables in the dataset. We assessed the relationship between each individual variable and the presence of overall POC. The results of this univariable analysis were used to select the most appropriate variables for inclusion in the multivariate analysis (*p* < 0.05). For multivariable analysis, permutation methods were applied to explore all possible combinations of predictors and select the best-performing logistic regression model. This permutation procedure was implemented using R's carret package. Different combinations of variables were evaluated, and the best-fitting model was selected using performance metrics such as the Akaike information criterion (AIC) and quality of fit.

All analyzes were performed using R version 4.3 and the dplyr, proc and carret packages.

### Supplementary Information

Supplementary Information.

## Data Availability

The datasets used and/or analysed during the current study available from the corresponding author on reasonable request.
