# Supplementary material for: Assessing the perioperative gain of weight (Δweight) as a determinant of morbidity after kidney transplantation: a retrospective exploratory study
Source: Sci Rep. 2024 Jun 11;14:13384. doi: 10.1038/s41598-024-63950-8 (PMC11167037; doi:10.1038/s41598-024-63950-8)
Supplement: Supplementary file 1 — Supplementary Information. [file 41598_2024_63950_MOESM1_ESM.docx]

**Supplementary Table 1: Uni- and multi-variable analysis for severe complications**

|  | **Univariable** | | | **Multivariable** | | |
| --- | --- | --- | --- | --- | --- | --- |
|  | **OR** | **95% CI** | **p-value** | **OR** | **95% CI** | **p-value** |
| Age (years) | 1.01 | 0.99-1.03 | 0.192 |  |  |  |
| Gender (female) | 0.79 | 0.44-1.41 | 0.423 |  |  |  |
| ASA score (<2) | 0.54 | 0.21-1.41 | 0.189 |  |  |  |
| Smoking | 1.12 | 0.63-1.99 | 0.7 |  |  |  |
| Diabetes (no diabetes) | 0.46 | 0.13-1.68 | 0.209 |  |  |  |
| Hypertension | 1.55 | 0.71-3.37 | 0.272 |  |  |  |
| BMI (kg/m^2^) | 1.05 | 0.99-1.11 | 0.09 |  |  |  |
| Type of graft (deceased-donor) | 1.51 | 0.87-2.64 | 0.143 |  |  |  |
| Duration of surgery (min) | 1.00 | 1.00-1.01 | 0.045 | 1.01 | 1.00-1.01 | 0.042 |
| ΔWeight (≥ 8.5 Kg) | 1.07 | 0.99-1.16 | 0.105 |  |  |  |
| ΔCreat (µmol/L) | 1.00 | 1.00-1.00 | 0.032 | 1.00 | 1.00-1.00 | 0.03 |

Abbreviations: OR: odd ratio; CI: confidence interval; ASA: American Society of Anesthesiologists; BMI: Body Mass Index
